# Comparison of survival outcomes between radio-chemotherapy and radical hysterectomy with postoperative standard therapy in patients with stage IB1 to IIA2 cervical cancer: long-term oncological outcome analysis in 37 Chinese hospitals

**DOI:** 10.1186/s12885-020-6651-8

**Published:** 2020-03-06

**Authors:** Ping Liu, Lihong Lin, Yanxiang Kong, Zhifeng Huo, Lin Zhu, Xiaonong Bin, Jinghe Lang, Chunlin Chen

**Affiliations:** 1 Department of Obstetrics and Gynecology, Nanfang Hospital, Southern Medical University, Guangzhou, 510515 China; 2 Department of Obstetrics and Gynecology, The Anyang Tumor Hospital of Henan Province, Anyang, 455000 China; 3 Department of Obstetrics and Gynecology, The Second Hospital of Shandong University, Jinan, 250033 China; 4 Department of Epidemiology, College of Public Health, Guangzhou Medical University, Guangzhou, 511436 China; 5 Department of Obstetrics and Gynecology, Peking Union Medical College Hospital, Peking Union Medical College, Beijing, 100730 China

**Keywords:** Cervical cancer, Radio-chemotherapy, Radical hysterectomy, Overall survival, Disease-free survival

## Abstract

**Background:**

This study aimed to compare the survival outcomes of radio-chemotherapy (R-CT) and radical hysterectomy with postoperative standard therapy (RH) in stage IB1-IIA2 cervical cancer patients.

**Methods:**

Based on the large amount of diagnostic and treatment cervical cancer data in China, a real-world study and 1:1 case-control matching were used to compare overall survival (OS) and disease-free survival (DFS) in cervical cancer patients.

**Results:**

In this real-world study, the 5-year OS and DFS in the R-CT group (*n* = 8949) were lower than those in the RH group (*n* = 18,152). After applying the inclusion criteria, the OS and DFS in the R-CT group (*n* = 582) were lower than those in the RH group (*n* = 4308). After 1:1 case-control matching, the 5-year OS and DFS in the R-CT group (*n* = 535) were lower than those in the RH group (*n* = 535) (OS: 76.1% vs. 84.6%, *p* < 0.001, HR = 1.819; DFS: 75.1% vs. 81.5%, *p* < 0.001, HR = 1.462, respectively). Further stratification showed that for stage IB1 and IIA1 patients, the 5-year OS and DFS in the R-CT group (*n* = 300) were lower than those in the RH group (*n* = 300) (OS: 78.9% vs. 87.0%, *p* < 0.001, HR = 2.160; DFS: 77.0% vs. 84.9%, *p* < 0.001, HR = 2.053, respectively). In stage IB2 and IIA2 patients, the 5-year OS in the R-CT group (*n* = 235) was lower than that in the RH group (*n* = 235) (72.5% vs. 81.5%, *p* = 0.039; HR = 1.550), but no difference in the 5-year DFS was found between the two groups (72.6% vs. 76.9%, *p* = 0.151).

**Conclusions:**

Our study found that for stage IB1-IIA2 cervical cancer patients, RH offers better overall survival and disease-free survival outcomes than R-CT, however, due to the inherent biases of retrospective study, it needs to be confirmed by randomized trials. In addition, we need to further understand the quality of life of the two treatments.

**Trial registration:**

registration number: CHiCTR1800017778; International Clinical Trials Registry Platform Search Port, http://apps.who.int/trialsearch/. registration date: August 14, 2018.

## Background

Cervical cancer is a common malignant tumour of the female genital tract and the fourth leading cause of cancer death among women worldwide, especially in developing countries. In 2018, there were 569,847 new cases worldwide and 311,365 deaths [1]. There are an estimated 98,900 new cases of cervical cancer in China each year and 30,500 deaths, accounting for 19 and 12% of the global data, respectively [2]. Treatment for cervical cancer includes radical hysterectomy, radiation therapy and chemotherapy. According to the 2019 National Comprehensive Cancer Network (NCCN) guidelines, radical hysterectomy + pelvic lymph node dissection (category 1), or radiotherapy/synchronized chemoradiotherapy can be used for stage IB1 and IIA1 patients, while for stage IB2 and IIA2 patients, definitive pelvic external beam radiation therapy (EBRT) + concurrent platinum-containing chemotherapy + brachytherapy (total point A dose ≥85 Gy) (category 1 for primary chemoradiation) or radical hysterectomy (category 2B) can be used [3].

Investigations into the therapeutic effects of different treatments on cervical cancer have not yielded consistent results [4]. In 2017, Landoni F found that the survival outcomes of radical hysterectomy and radiotherapy were similar in a prospective single-centre study of 20 years on IB1-IIA2 cervical cancer patients [5]. Some studies have also concluded that the survival outcomes of radical hysterectomy in stage IB1-IIA2 squamous cell carcinoma are similar to those of radiotherapy [4, 6–8]. However, two Surveillance, Epidemiology, and End Results (SEER) studies from America suggest that surgical treatment significantly improves survival outcomes in patients with stage IB1-IIA2 cervical cancer [9, 10]. There is also debate regarding the therapeutic effects of different treatments in patients with different stages of cervical cancer. Unfortunately, the above studies lack data from developing countries.

China has a large amount of data on cervical cancer, which has important reference value. Therefore, we conducted a real-world study in cooperation with 37 hospitals in China that independently perform radical hysterectomy procedures. From 2004 to 2016, the clinical data of all hospitalized cervical cancer patients were collected comprehensively, carefully and completely, and the long-term oncological outcomes of the patients were followed. A large database of clinical diagnoses and treatments for cervical cancer in China was constructed. After screening IB1-IIA2 cervical cancer cases from the database, we compared the oncological outcomes of radio-chemotherapy (R-CT) and radical hysterectomy (RH) with postoperative standard therapy to explore their therapeutic effects on patients in China.

## Methods

### Establishment of the China cervical Cancer clinical database

#### Data collection

This retrospective study was approved by the Ethics Committee of the Nanfang Hospital of Southern Medical University (approval number NFEC-2017-135 and clinical trial number CHiCTR1800017778; International Clinical Trials Registry Platform Search Port, http://apps.who.int/trialsearch/). All staff who handled patient data were trained on the hospital’s medical record management system to transfer all hospitalized cervical cancer patients from 2004 to 2016. The input indicators included general patient data, related surgical data, disease-related test results, postoperative pathology results, adjuvant treatment data, and follow-up data. After the entries were completed, two gynaecologists performed independent information checks to ensure accuracy. We used the International Federation of Gynecology and Obstetrics (FIGO) clinical staging system to classify the cancer stage. Due to the large time period included in the study, cases from 2004 to 2009 were adjusted in accordance with the 2009 FIGO guidelines [11–14]. Any missing or incomplete data in a given medical record were supplemented according to the patient’s specific examination record, imaging record, colposcopy record, postoperative pathological record, etc. The pathological types included squamous cell carcinoma, adenocarcinoma and adenosquamous carcinoma [12, 13]. The remaining information was obtained from medical document files, such as pathology reports, surgical records, and discharge records.

#### Follow-up and data management

To ensure the privacy of all patients, all follow-up procedures were conducted by trained gynaecologists and monitored by specified staff. Through follow-up phone calls, we were able to review the information on survival, recurrence status and complications. We also reminded every patient to undergo routine physical examinations. If a patient could not be reached by telephone, a thorough search of the outpatient system, picture archiving and communication system (PACS), and clinical laboratory information system was conducted. The latest records were considered the time to survival. In addition, information regarding recurrence was extracted through outpatient medical records.

#### Data double input

To ensure the accuracy of data entry, two specially trained gynaecologists double-entered the same medical record, and any suspected parameters were checked and entered into the database.

#### Data storage

After entering all case information and follow-up data and completing double-input verification, the patient data were summarized and managed by a professional to establish a unified database.

### Case screening criteria for this study

#### Inclusion criteria

We selected cases according to the following criteria:

1. R-CT group: age ≥ 18 years old; clinical stage IB1-IIA2; histological type of squamous cell carcinoma, adenocarcinoma or adenosquamous carcinoma; initial treatment with R-CT; treatment including external irradiation + afterloading; radiotherapy dose higher than 40 Gy; chemotherapy regimens including paclitaxel + carboplatin, paclitaxel + other platinum, platinum +5FU, platinum + other, etc., which were used according to guidelines and drug instructions; survival outcome information available; and all patients able to complete the treatment.
2. RH with postoperative standard therapy group (RH group): age ≥ 18 years old; clinical stage IB1-IIA2; histological type of squamous cell carcinoma, adenocarcinoma or adenosquamous carcinoma; initial treatment of open surgery, QM-B or QM-C hysterectomy + pelvic lymphadenectomy ± para-aortic lymph node resection; no neoadjuvant chemotherapy or radiotherapy; postoperative standard adjuvant treatment according to the pathological factors described by the guidelines [3, 15] (for example, pelvic external irradiation + cisplatin combined with chemotherapy ± vaginal brachytherapy would be performed if there were one or more postoperative pathological risk factors (lymph node positive, incisal margin involvement or para-uterine involvement); pelvic external irradiation ± concurrent chemotherapy containing cisplatin if two or more postoperative pathological risk factors were noted (tumour diameter ≥ 4 cm, cervical invasion depth ≥ 1/2 and LVSI invasion)); treatment including external irradiation + afterloading; radiotherapy dose higher than 40 Gy; chemotherapy regimens including paclitaxel + carboplatin, paclitaxel + other platinum, platinum +5FU, platinum + other, etc., which were used according to guidelines and drug instructions; and available survival outcome information.

#### Exclusion criteria

The exclusion criteria were as follows:

(1) R-CT group: FIGO stage unknown, not standard, or staged in stage IIB or higher; pregnancy with cervical cancer; accidental discovery of cervical cancer; stump cancer or other malignant tumours; radiotherapy dose record unknown or simple external irradiation; and no survival outcome information available. (2) RH group: FIGO stage unknown, not standard, or staged above stage IIB; no surgical treatment, except for QM-B or QM-C hysterectomy; other types of RH; preoperative neoadjuvant chemotherapy or radiotherapy; postoperative adjuvant radiotherapy dose was not recorded or radiotherapy was not available; no pelvic lymphadenectomy or pelvic lymph node resection unknown; pregnancy with cervical cancer; accidental discovery of cervical cancer; stump cancer; patients with other types of malignant tumours; and no survival outcome information available.

### Observation indicators

The primary outcomes were overall survival (OS) and disease-free survival (DFS), and five years was the cut-off point for long-term oncologic outcomes.

### Statistical methods

Data analysis was performed using SPSS statistical software (version 23.0, SPSS Inc., Chicago, IL, USA). The measurement data are expressed as the mean ± standard deviation (x ± s), and the count data are expressed as a percentage (%). For continuous data, the normality test was first performed. If each group satisfied the normality condition and the variance between the two groups was either equal or not equal, a t-test was used for comparisons between groups; otherwise, the non-parametric rank sum test was considered. For classified data, the chi-square test was used for disordered outcomes, and the non-parametric rank sum test was used for ordered data. One-to-one case-control matching was used to adjust the baseline data, with the case-control matching parameter settings as follows: the match indicator age tolerance was 3, and the other index tolerance was 0; sample matching was performed without replacement; cases were randomly sequenced during extraction with priority matching sampling; and the seed number was 123,456. In this study, Kaplan-Meier curves were used to describe changes in survival, and log-rank tests were used to compare the differences in the survival curves. For multivariate analysis, the treatment plan, year, FIGO stage, histological type, tumour diameter and age were included. If the proportional risk assumption was met, Cox regression analysis was used to correct the effects of other confounding factors on survival. If the proportional hazard assumption was not met, then the effects of the non-equal Cox regression analysis of the study factors were considered. The hazard ratio was calculated for only the variables included in the Cox regression model, and the factors not included in the model had no corresponding hazard ratios; *p* < 0.05 was considered significant. One-to-one case-control matching was performed based on the patient’s age, FIGO stage, histological type, and tumour diameter.

## Results

### Data screening process

**Fig. 1:**
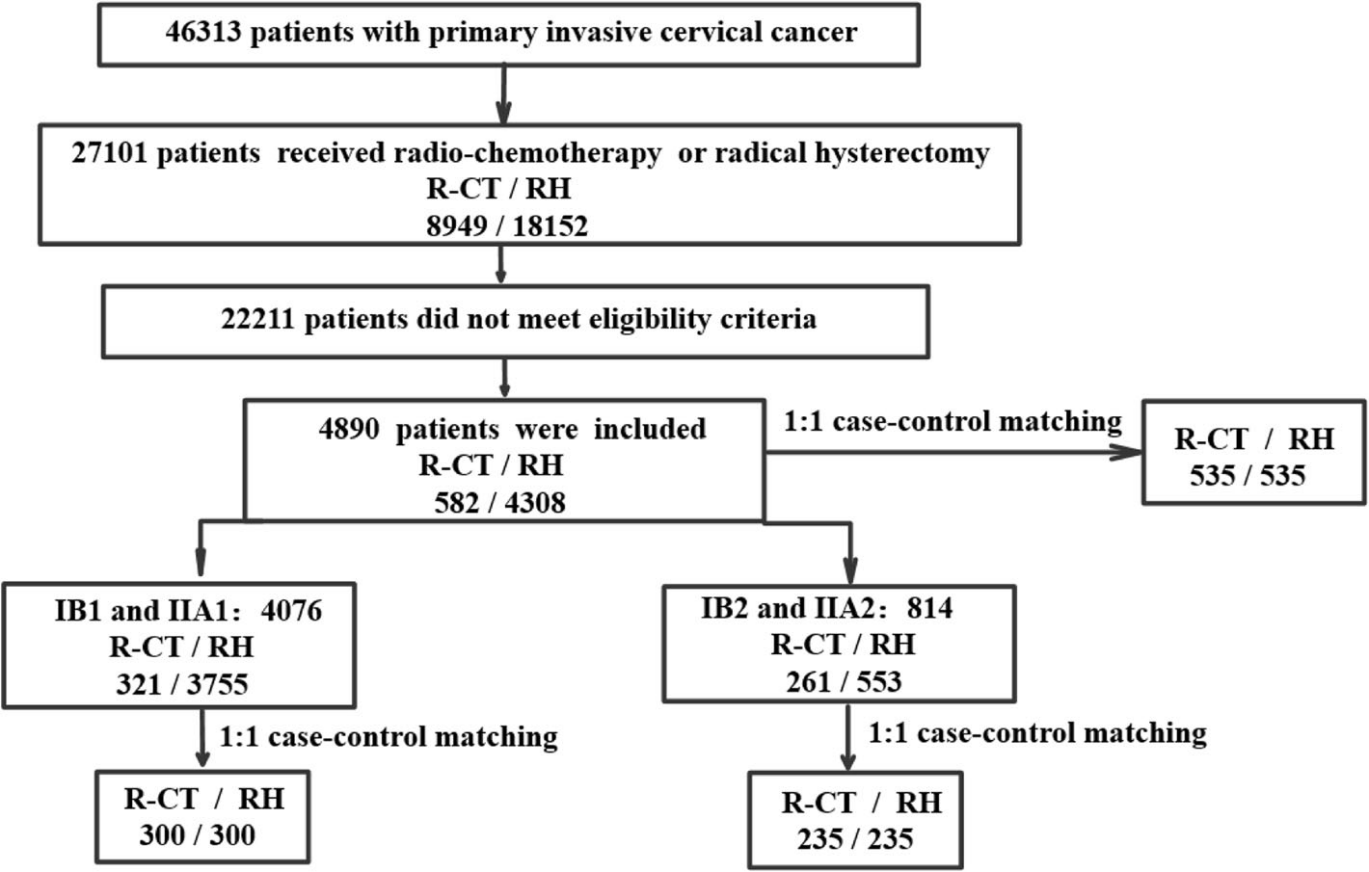
Data screening process. R-CT: radio-chemotherapy, RH: radical hysterectomy with postoperative standard therapy

Of the 46,313 patients who were enrolled, 8949 patients were assigned to the R-CT group, and 18,152 patients were assigned to the RH group for this real-world study. According to the screening criteria, 582 patients were assigned to the R-CT group, and 4308 patients were assigned to the RH group. After matching, a total of 535 patients were included in the two groups. Finally, according to different stages, 321 patients with stage IB1 and IIA1 cervical cancer were assigned to the R-CT group, and 3755 patients were assigned to the RH group. Three hundred patients were included after matching. For stage IB2 and IIA2, 261 patients were assigned to the R-CT group, and 553 patients were assigned to the RH group. A total of 235 patients were included after matching. The data screening process is shown in Fig. 1.

### Differences in survival outcomes between the radio-chemotherapy group and the radical hysterectomy group

In the R-CT group (*n* = 8949) and RH group (*n* = 18,152), the median follow-up was 34 months and 51 months, respectively, and the number of deaths in 5 years was 1503 (16.8%) and 948 (10.6%), respectively; the 5-year OS was 69.3% vs. 91.1% (*p* < 0.001); and the DFS was 65.0% vs. 86.7% (*p* < 0.001), respectively. Cox multivariate analysis showed a higher risk of death or recurrence/death in the R-CT group than in the RH group (death: HR = 3.628, *p* < 0.001; recurrence/death: HR = 3.160, *p* < 0.001).

### Differences in survival outcomes between the radio-chemotherapy group and the radical hysterectomy with postoperative standard therapy group

The baseline distribution of FIGO stage, histological type, tumour diameter, and age was not balanced among the 4890 patients who were included. To reduce the influence of confounding factors, we performed 1:1 case-control matching and then performed a survival analysis.

Before matching, the median follow-up in the R-CT group (*n* = 582) and RH group (*n* = 4308) was 34 months and 47 months, respectively; the number of deaths in 5 years was 108 (18.6%) and 292 (6.8%), respectively; the 5-year OS was 75.0% vs. 91.5% (*p* < 0.001), and the DFS was 72.9% vs. 86.6% (*p* < 0.001), respectively. Cox multivariate analysis showed a higher risk of death or recurrence/death in the R-CT group than in the RH group (HR = 2.187, *p* < 0.001 vs. HR = 1.661, *p* < 0.001).

**Fig. 2:**
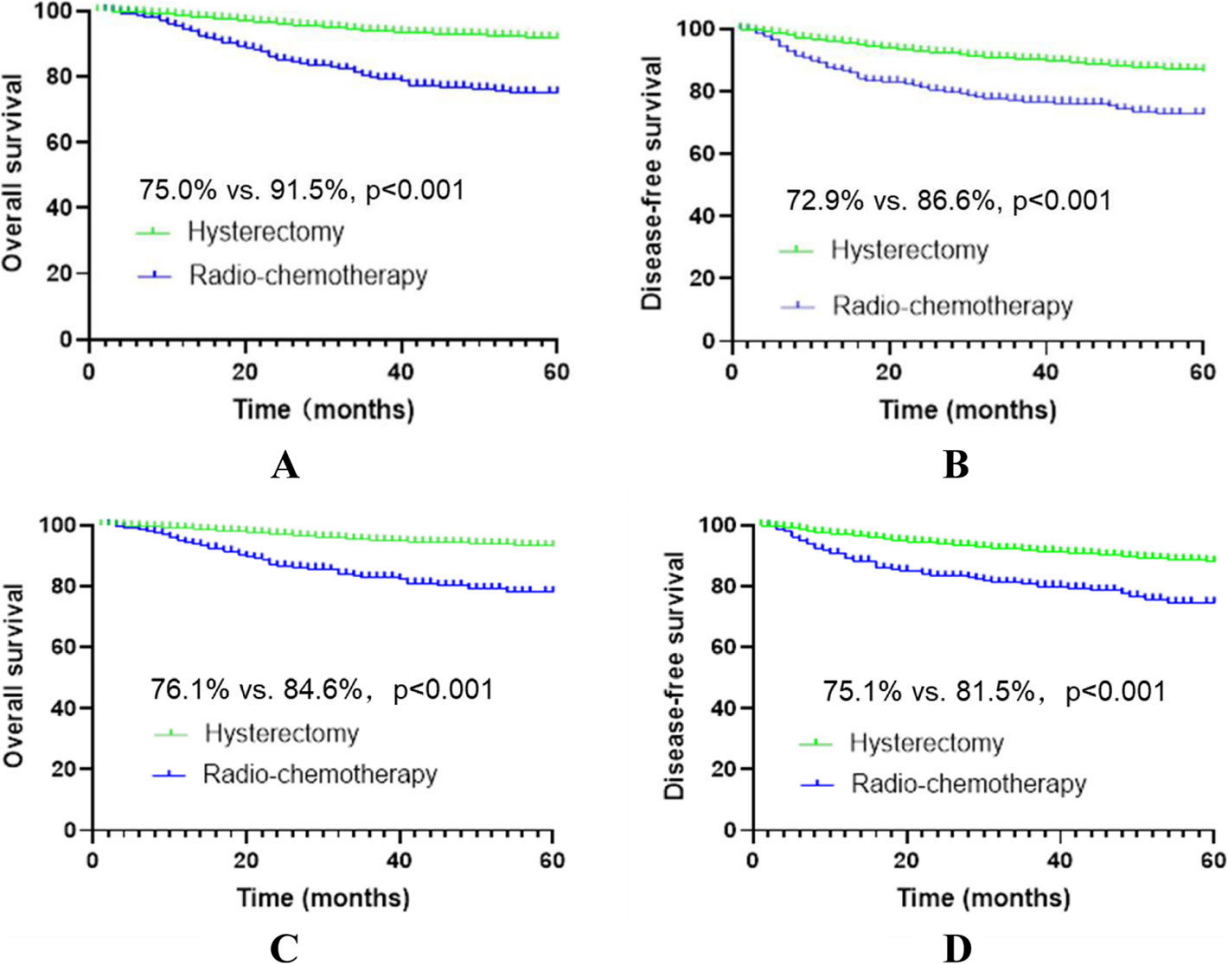
Survival curves before and after matching stage IB1 to IIA2 cervical cancer patients who met the study criteria. *Before matching, panels **a** and **b**; after matching, panels **c** and **d**

After matching, 535 patients were included in each group. The median follow-up was 34 months and 46 months in the R-CT group and the RH group, respectively; the number of deaths was 94 (17.6%) and 65 (12.1%), respectively; the 5-year OS was 76.1% vs. 84.6% (*p* < 0.001), respectively; and the DFS was 75.1% vs. 81.5%, *p* < 0.001, respectively. Cox multivariate analysis showed a higher risk of death or recurrence/death in the R-CT group than in the RH group (HR = 1.819, *p* < 0.001 vs. HR = 1.462, *p* < 0.001) (Table 1, Fig. 2).

**Table 1:** Data of stage IB1 to IIA2 patients before and after matching

Variables	Unmatched		Matched			
	R-CT (*n* = 582)	RH (*n* = 4308)	*p*-value	R-CT (*n* = 535)	RH (*n* = 535)	*p*-value
Age (years)	55.4 ± 11.2	48.0 ± 9.7	0.000	54.3 ± 10.1	54.3 ± 10.0	1.000
FIGO stage			0.000			1.000
IB1	84 (14.4%)	2770 (64.3%)		81 (15.1%)	81 (15.1%)	
IB2	60 (10.3%)	322 (7.5%)		56 (10.5%)	56 (10.5%)	
IIA1	237 (40.7%)	985 (22.9%)		219 (40.9%)	219 (40.9%)	
IIA2	201 (34.6%)	231 (5.3%)		179 (33.5%)	179 (33.5%)	
Histological type			0.000			1.000
SCC	549 (94.3%)	3863 (89.7%)		516 (96.4%)	516 (96.4%)	
AC	20 (3.5%)	341 (7.9%)		11 (2.1%)	11 (2.1%)	
SAC	13 (2.2%)	104 (2.4%)		8 (1.5%)	8 (1.5%)	
Tumour size			0.000			1.000
> 4 cm	292 (50.2%)	3404 (79.0%)		268 (50.1%)	268 (50.1%)	
≤ 4 cm	290 (49.8%)	904 (21.0%)		267 (49.9%)	267 (49.9%)	

*R-CT* radio-chemotherapy, *RH* radical hysterectomy with postoperative standard therapy, *FIGO* International Federation of Gynecology and Obstetrics, *SCC* squamous cell carcinoma, *AC* adenocarcinoma, *SAC* adenosquamous carcinoma

### Differences in survival outcomes between the two groups before and after matching: patients with stage IB1 and IIA1 cervical cancer

In patients with stage IB1 and IIA1 cervical cancer, the baseline between the R-CT group (*n* = 321) and the RH group (*n* = 3755) was unbalanced, and 300 patients were included in the matched group. There was no significant difference between the two groups.

Before matching, the median follow-up was 35 months and 47 months in the R-CT group and the RH group, respectively; the number of deaths was 53 (16.6%) and 207 (5.5%), respectively; the 5-year OS was 78.0% vs. 93.0% (*p* < 0.001), respectively; and the DFS was 74.6% vs. 87.9% (*p* < 0.001), respectively. Cox multivariate analysis showed a higher risk of death or recurrence/death in the R-CT group than in the RH group (HR = 2.703, *p* < 0.001 vs. HR = 1.843, *p* < 0.001).

**Fig. 3:**
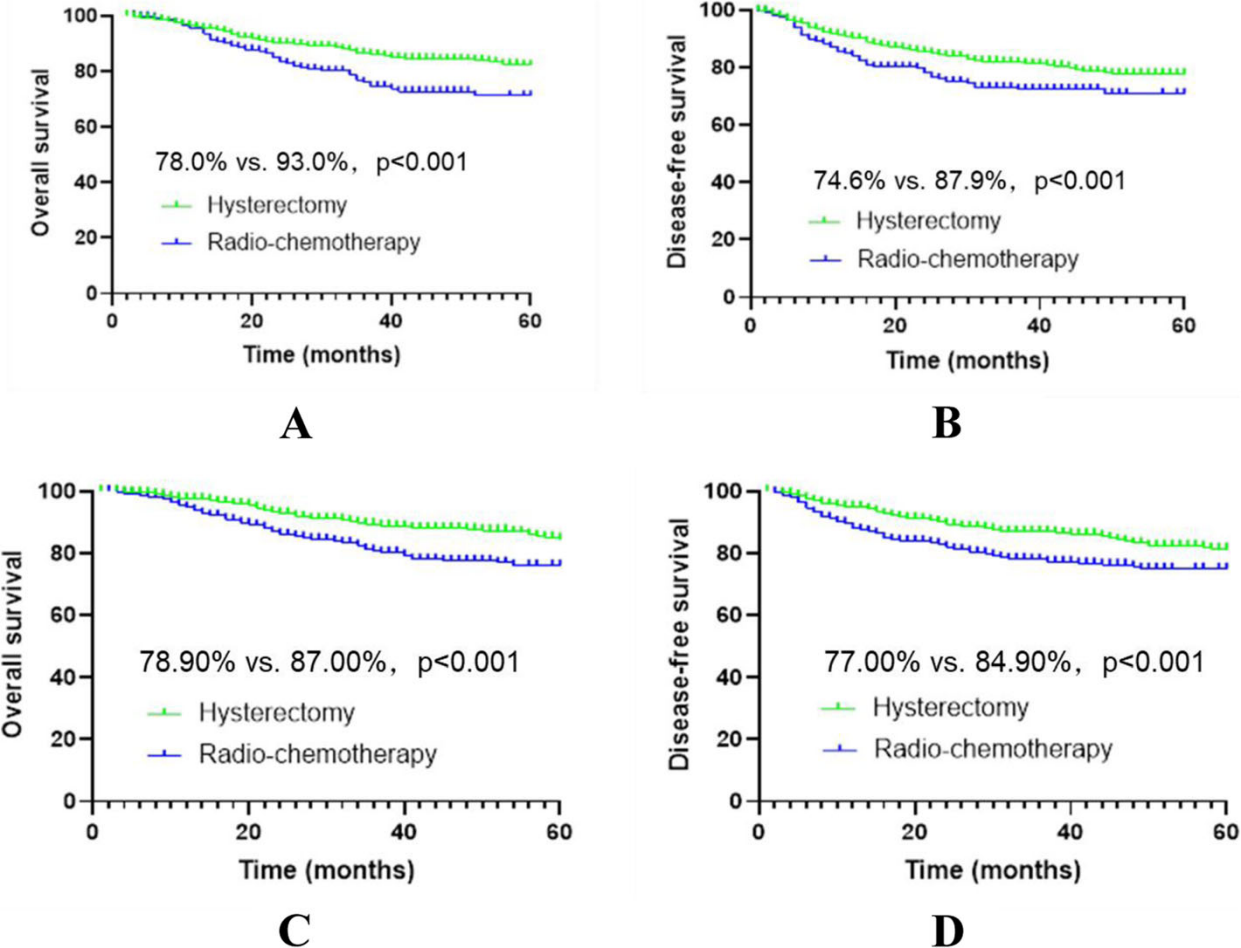
Survival curves of IB1 and IIA1 cervical cancer patients before and after matching. *Before matching, panels **a** and **b**; after matching, panels **c** and **d**

After matching, the median follow-up was 35 months and 43 months in the R-CT group and RH group, respectively; the number of deaths was 48 (16.0%) and 27 (9.0%), respectively; the 5-year OS was 78.9% vs. 87.0% (*p* < 0.001), respectively; and the DFS was 77.0% vs. 84.9% (*p* < 0.001), respectively. Cox multivariate analysis showed a higher risk of death or recurrence/death in the R-CT group than in the RH group (HR = 2.160, *p* < 0.001 vs. HR = 2.053, *p* < 0.001) (Table 2, Fig. 3).

**Table 2:** Data of stage IB1 and IIA1 patients before and after matching

Variables	Unmatched		Matched			
	R-CT (*n* = 321)	RH (*n* = 3755)	*p*-value	R-CT (*n* = 300)	RH (*n* = 300)	*p*-value
Age (years)	58.9 ± 10.8	48.0 ± 9.8	0.000	57.7 ± 10.0	57.5 ± 9.8	0.874
FIGO stage			0.000			1.000
IB1	84 (26.2%)	2770 (73.8%)		81 (27.0%)	81 (27.0%)	
IIA1	237 (73.8%)	985 (26.3%)		219 (73.0%)	219 (73.0%)	
Histological type			0.000			1.000
SCC	308 (96.0%)	3350 (89.2%)		288 (96.0%)	288 (96.0%)	
AC	9 (2.8%)	310 (8.3%)		8 (2.7%)	8 (2.7%)	
SAC	4 (1.2%)	95 (2.5%)		4 (1.3%)	4 (1.3%)	
Tumour size			0.000			1.000
> 4 cm	60 (18.7%)	470 (12.5%)		58 (19.3%)	58 (19.3%)	
≤ 4 cm	261 (81.3%)	3285 (87.5%)		242 (80.7%)	242 (80.7%)	

*R-CT* radio-chemotherapy, *RH* radical hysterectomy with postoperative standard therapy, *FIGO* International Federation of Gynecology and Obstetrics, *SCC* squamous cell carcinoma, *AC* adenocarcinoma, *SAC* adenosquamous carcinoma

### Differences in survival outcomes between the two groups before and after matching: patients with stage IB2 and IIA2 cervical cancer

In patients with stage IB2 and IIA2 cervical cancer, the baseline between the R-CT group (*n* = 261) and the RH group (*n* = 553) was not balanced, and 235 patients were included in the matched group. There was no significant difference between the two groups.

Before matching, the median follow-up was 31 months and 48 months in the R-CT group and RH group, respectively; the number of deaths was 55 (21.1%) and 85 (15.4%), respectively; the 5-year OS in the R-CT group vs. the RH group was 71.2% vs. 82.1% (*p* < 0.001), respectively; and the DFS was 71.1% vs. 77.8% (*p* < 0.001), respectively. Cox multivariate analysis showed a higher risk of death or recurrence/death in the R-CT group than in the RH group (HR = 1.720, *p* < 0.001 vs. HR = 1.752, *p* < 0.001).

**Fig. 4:**
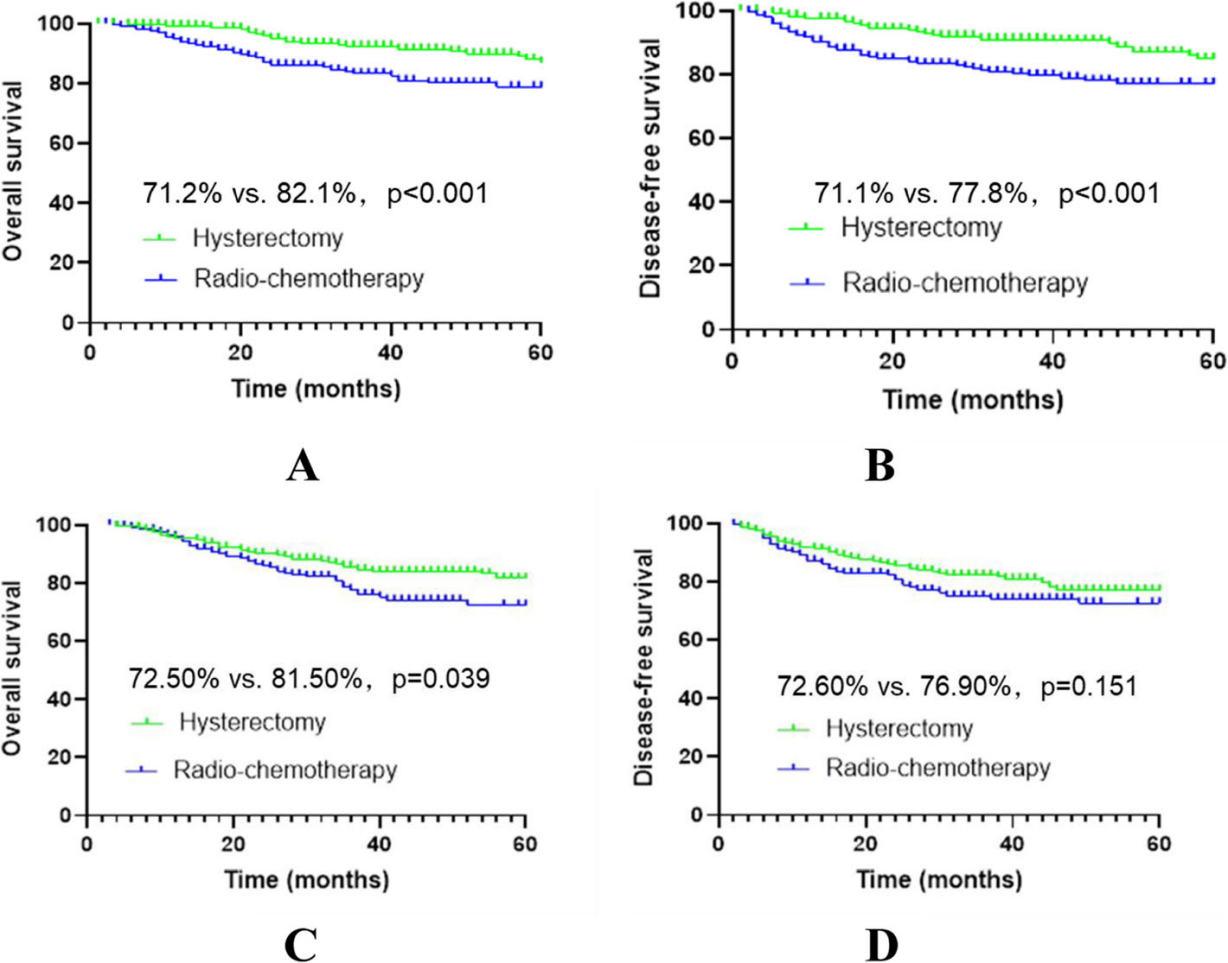
Survival curves of stage IB2 and IIA2 cervical cancer patients before and after matching. *Before matching, panels **a** and **b**; after matching, panels **c** and **d**

After matching, the median follow-up was 32 months and 49 months in the R-CT group and RH group, respectively; the number of deaths was 46 (19.6%) and 38 (16.2%), respectively, and the number of deaths or recurrences was 53 (22.6%) and 49 (20.9%), respectively; the 5-year OS was 72.5% vs. 81.5% (*p* = 0.039), respectively; and the DFS was 72.6% vs. 76.9% (*p* = 0.151), respectively. Cox multivariate analysis showed a higher risk of death in the R-CT group than in the RH group (HR = 1.550, *p* = 0.047). No significant difference was found between the two groups in terms of recurrence/death risk (*p* = 0.146) (Table 3, Fig. 4).

**Table 3:** Data of patients with stage IB2 and IIA2 cervical cancer before and after matching

Variables	Unmatched		Matched			
	R-CT (*n* = 261)	RH (*n* = 553)	*p*-value	R-CT (*n* = 235)	RH (*n* = 235)	*p*-value
Age (years)	51.2 ± 10.4	47.6 ± 8.7	0.000	50.4 ± 8.8	50.3 ± 8.7	0.924
FIGO stage			0.000			1.000
IB2	60 (23.0%)	322 (58.2%)		56 (23.8%)	56 (23.8%)	
IIA2	201 (77.0%)	231 (41.8%)		179 (76.2%)	179 (76.2%)	
Histological type			0.000			1.000
SCC	241 (92.3%)	513 (92.8%)		228 (97.0%)	228 (97.0%)	
AC	11 (4.2%)	31 (5.6%)		3 (1.3%)	3 (1.3%)	
SAC	9 (3.5%)	9 (1.6%)		4 (1.7%)	4 (1.7%)	
Tumour size			0.000			1.000
> 4 cm	230 (88.1%)	434 (78.5%)		210 (89.4%)	210 (89.4%)	
≤ 4 cm	31 (11.9%)	119 (21.5)		25 (10.6%)	25 (10.6%)	

*R-CT* radio-chemotherapy, *RH* radical hysterectomy with postoperative standard therapy, *FIGO* International Federation of Gynecology and Obstetrics, *SCC* squamous cell carcinoma, *AC* adenocarcinoma, *SAC* adenosquamous carcinoma

## Discussion

In our study, the surgical approach in the RH group was laparotomy to rule out deviation. In 2018, Ramirez et al. reported in the New England Journal of Medicine that, in women with early cervical cancer, the DFS and OS rates after minimally invasive radical hysterectomy were lower than those after open radical hysterectomy [16]. In our research, RH accounted for 91.63% (3755/4098) of stage IB1 and IIA1 cases and 67% (553/825) of stage IB2 and IIA2 cases of cervical cancer. The 2019 NCCN guidelines recommend that RH + pelvic lymph node dissection (category 1) or radiotherapy/synchronized chemoradiotherapy can be used for stage IB1 and IIA1 patients, while for stage IB2 and IIA2 patients, definitive pelvic EBRT + concurrent platinum-containing chemotherapy + brachytherapy (total point A dose ≥85 Gy) (category 1 for primary chemoradiation) or RH (category 2B) can be used [3, 17]. However, in China, RH is still the main treatment for early cervical cancer.

Many previous studies have shown that the outcomes of R-CT are similar to those of RH [5, 8, 18]. In 2017, Landoni F et al. [5] conducted a prospective single-centre study that was initiated in 1997 for a follow-up of 20 years and concluded that the outcomes of radiotherapy and RH were similar in stage IB1-IIA2 cervical cancer patients. In 2017, Wu S et al. [19] found no difference in the survival outcomes of stage IB1 and IIA1 cervical cancer patients. However, some studies have suggested that surgical treatment is superior to R-CT. In 2009, Bansal N et al. [9] analysed stage IB1-IIA2 cervical cancer in the SEER database and found that for women with tumours smaller than 6 cm, surgical treatment is superior to radiotherapy. In 2012, Rungruang B [10] analysed only patients with stage IB2 cervical cancer and concluded that the total survival time of the RH group was longer than that of the radiotherapy group.

In our real-world study, RH offered superior oncologic outcomes. According to the inclusion criteria, the oncological outcome of the RH with postoperative standard treatment group was superior to that of the R-CT group. After controlling for confounding factors, the results still showed that the oncological outcome of the RH with postoperative standard treatment group was superior to that of the R-CT group. The 5-year OS was 76.1% vs. 84.6%, and the DFS was 75.1% vs. 81.5% in the R-CT group and the RH group, respectively. Cox multivariate analysis showed that the R-CT group had a higher risk of death or recurrence/death than the RH group (HR = 1.819, *p* < 0.001; HR = 1.462, *p* < 0.001).

Further analysis was performed according to different stages. For stage IB1 and IIA1 patients, the R-CT group had worse oncologic outcomes than the RH group both before and after matching (before matching: OS: 78.0% vs. 93.0%, respectively, *p* < 0.001, HR = 2.703; DFS: 74.6% vs. 87.9%, respectively, *p* < 0.001, HR = 1.843; after matching: OS: 78.90% vs. 87.00%, respectively, *p* < 0.001, HR = 2.160; DFS: 77.00% vs. 84.90%, respectively, *p* < 0.001, HR = 2.053). For stage IB2 and IIA2 patients, the 5-year OS of the R-CT group was lower than that of the RH group before matching (72.50% vs. 81.50%, respectively, *p* = 0.039; HR = 1.550). No difference in the 5-year DFS was observed between the two groups (72.60% vs. 76.90%, *p* = 0.151).

The results of this paper are not completely consistent with those of Landoni F, Newton M, Yamashita H and Wu S [4, 5, 7, 18, 19] but are similar to the findings of Bansal N and Rungruang B [9, 10]. The reasons may be as follows. (1) The number of included cases differed across studies. As the number of cases in Newton M and Landoni F was 124–343, the differences between the groups may not be accurately reflected. In contrast, Bansal N and Rungruang B analysed 4885 cases (4012 RH and 873 radiotherapy) and 770 cases (401 RH and 369 radiotherapy), respectively; our results are similar to the findings in these articles. 2) Newton M, Yamashita H, Wu S and the other studies did not consider postoperative adjuvant therapy. As 64% of patients in the Landoni et al. study underwent adjuvant RT, this difference could represent a bias. The patients in our study were standardized according to the NCCN guidelines and a relevant study [3, 15] in which postoperative standard treatment should be performed based on pathological factors that contribute to improved oncological outcomes. In our study, 42.3% of the patients in the RH group underwent postoperative adjuvant treatment; 6.3% received radiotherapy alone, 8.7% received concurrent radiotherapy and chemotherapy, and 27.3% received radiotherapy plus chemotherapy. Moreover, compared with previous studies, in the present study, the effects of unknown confounding factors were reduced, so the results can be considered more credible.

Compared with RH, radiotherapy can lead to ovarian failure and potential radiation-related complications, such as radiation cystitis, proctitis, fistula formation, vaginal shortening and dryness, and impaired sexual function [5], seriously affecting patient quality of life. In summary, we believe that radical hysterectomy ± postoperative standard adjuvant therapy should be recommended to patients with stage IB1-IIA2 cervical cancer.

This study has the following limitations. The diameter of the tumour was only classified as 4 cm according to the guidelines; there was no subdivision with regard to tumour size. However, after matching and adjusting for confounding factors, Cox multivariate analysis showed that the tumour diameter was not a relevant factor affecting the oncological outcome. Second, radiotherapy + chemotherapy was included in the R-CT group (some Chinese physicians also call this regimen “sequential therapy”). Radiotherapy + chemotherapy has certain applications in diagnosis and treatment in China. The inclusion of radiotherapy and chemotherapy can more objectively and accurately reflect the current diagnosis and treatment of cervical cancer in China.

## Conclusions

Our study found that for stage IB1-IIA2 cervical cancer patients, radical hysterectomy with postoperative standard therapy (RH) offers better overall survival and disease-free survival outcomes than radio-chemotherapy (R-CT), however, due to the inherent biases of retrospective study, it needs to be confirmed by randomized trials. In addition, we need to further understand the quality of life of the two treatments.

## Data Availability

The datasets used and/or analysed during the current study are available from the corresponding author upon reasonable request.

## Abbreviations

AC: Adenocarcinoma
FIGO: International Federation of Gynecology and Obstetrics
R-CT: Radio-chemotherapy
RH: Radical hysterectomy with postoperative standard therapy
SAC: Adenosquamous carcinoma
SCC: Squamous cell carcinoma
